# Advancing the application of systems thinking in health: advice seeking behavior among primary health care physicians in Pakistan

**DOI:** 10.1186/1478-4505-12-43

**Published:** 2014-08-26

**Authors:** Asmat U Malik, Cameron D Willis, Saima Hamid, Anar Ulikpan, Peter S Hill

**Affiliations:** Integrated Health Services, House 1-B, Street 50, Sector F-8/4, Islamabad, Pakistan; Propel Centre for Population Health Impact, University of Waterloo, 200 University Avenue West, Waterloo, ON N2L 3G1 Canada; Health Services Academy, Prime Minister’s National Health Complex, Government of Pakistan, Park Road, Chak Shahzad, Islamabad, Pakistan; School of Population Health, The University of Queensland, Herston Road, 4006 Herston, QLD Australia

**Keywords:** Health system, Measles, Pakistan, Primary health care, Social network analysis, Systems thinking, Tuberculosis

## Abstract

**Background:**

Using measles and tuberculosis as case examples, with a systems thinking approach, this study examines the human advice-seeking behavior of primary health care (PHC) physicians in a rural district of Pakistan. This study analyzes the degree to which the existing PHC system supports their access to human advice, and explores in what ways this system might be strengthened to better meet provider needs.

**Methods:**

The study was conducted in a rural district of Pakistan and, with a cross-sectional study design, it employed a range of research methods, namely extensive document review for mapping existing information systems, social network analysis of physicians’ advice-seeking practice, and key stakeholder interviews for an in-depth understanding of the experience of physicians. Illustrations were prepared for information flow mechanism, sociographs were generated for analyzing social networks, and content analysis of qualitative findings was carried out for in-depth interpretation of underlying meanings.

**Results:**

The findings of this study reveal that non-availability of competent supervisory staff, a focus on improving performance indicators rather than clinical guidance, and a lack of a functional referral system have collectively created an environment in which PHC physicians have developed their own strategies to overcome these constraints. They are well aware of the human expertise available within and outside the district. However, their advice-seeking behavior was dependent upon existence of informal social interaction with the senior specialists. Despite the limitations of the system, the physicians proactively used their professional linkages to seek advice and also to refer patients to the referral center based on their experience and the facilities that they trusted.

**Conclusions:**

The absence of functional referral systems, limited effective linkages between PHC and higher levels of care, and a focus on programmatic targets rather than clinical care have each contributed to the isolation of physicians and reactive information seeking behavior. The study findings underscore the need for a functional information system comprising context sensitive knowledge management and translation opportunities for physicians working in PHC centers. Such an information system needs to link people and resources in ways that transcend geography and discipline, and that builds on existing expertise, interpersonal relationships, and trust.

## Background

Access to information is critical for creating and maintaining high performing primary health care (PHC) systems [1]. This aspect becomes especially important when frontline health workers (such as physicians, nurses, or allied health providers) face difficulties in diagnosing cases in PHC settings where consultations are of short duration [2], they are confronted with a wide range of medical problems [3], and their information needs are motivated by the specific needs of patient care [3, 4]. Davies [5] describes multiple sources of information that are available to physicians for assisting clinical decision making in difficult to diagnose cases, including clinical guidelines and research papers, as well as advice provided by other professionals [5] such as peers, fellow physicians, consultants, and teachers [6, 7].

Huth et al. [8] note that physicians often seek advice from human sources when they are looking for readily available and convenient sources of information [8], mostly related to diagnostics, management, and referral strategies [7]. Text books, research papers, and other sources of information may not be adequate to answer their questions as many times physicians are also searching for support, guidance, affirmation, and feedback [9], which requires a synthesis of medical knowledge, patient information, and an understanding of the context of care [4], especially in complex cases [10].

The available studies provide some insights into how physicians seek information while working in PHC settings [2, 5, 11]. However, as this literature is largely confined to high-income countries, there is relatively little known about how physicians in low- and middle-income countries access or use information from human sources when faced with difficult to diagnose conditions. In these settings, where access to electronic information sources is often scarce, an understanding about advice-seeking behavior from human sources becomes particularly important.

Applying a systems thinking lens to understanding advice-seeking behavior in Pakistan’s PHC system is a key component of the healthcare delivery system [12], with PHC physicians being main actors within a complex health system [13]. Their behavior, linkages, relationships, and interactions influence, and are influenced by, the system and its components [14]. Understanding and informing policy processes that are also influenced by human behaviors requires evidence that reflects the behavior of key actors, such as PHC physicians, and how these behaviors interact over time within social networks. Despite efforts to maintain consistency and uniformity in policy implementation through hierarchical control and command systems, there remain variations in how health professionals at the ‘street-level’ implement such guidance [15].

Systems thinking encourages a dynamic and inter-related perspective of system structure and function, emphasizing the importance of relationships between parts and whole, and the unpredictability of system behaviors [16, 17]. Sterman [18] describes systems thinking as an “*an iterative learning process in which we replace a reductionist, narrow, short-run, static view of the world with a holistic, broad, long-term, dynamic view, reinventing our policies and institutions accordingly*”. A systems thinking lens allows us to recognize the importance of long-term change, the power of context, the role of guiding principles (rather than prescriptive control), the centrality of knowledge, and the enabling contributions made by interpersonal and inter-organizational relationships as vehicles for knowledge translation and exchange [19]. It is therefore a powerful lens through which advice-seeking behavior may be understood.

Using methods grounded in systems science, this paper examines the human advice-seeking behavior of PHC physicians in a rural district of Pakistan in the public health sector, analyzing the degree to which the existing PHC system supports their access to human advice and exploring in what ways this system might be strengthened to better meet provider needs. It goes beyond an analysis of what information is sought by physicians – the common objective of needs-based studies – to understand how and from whom that information is sought. Using a systems lens, operationalized in part through social network analysis, explores the richness of interaction through both formal and non-formal relationships in the context of the PHC system and their implications for clinical decision making.

### Research questions

The specific research questions of this study are:

- To what degree does the existing structure of the PHC system in Pakistan support physicians in accessing advice from human sources on difficult to diagnose cases?
- To what degree are physicians satisfied with their current access to advice from human sources on difficult to diagnose cases?
- What changes, if any, do physicians recommend to improve their access to advice from human sources on difficult to diagnose cases?

## Methods

This study was conducted at the district level in Pakistan from January 2013 to August 2013. District Attock, predominantly a rural district with a population of 1.6 million, was conveniently selected as a case illustration because of its proximity to the principal investigator [20].

In order to align the study questions with the health problems that the district health department considered a priority, targeted key informant interviews were conducted with five district health administrators^a^ and line-managers^b^ of vertical health programs who were purposively selected on the basis of their extensive knowledge of the information systems and their experience of working in district health systems. Key informants were specifically asked to nominate two priority health problems for use as case studies in order to map information flow mechanisms and analyze the advice-seeking behavior of physicians working in Basic Health Units (BHU). Tuberculosis (TB) and measles were identified as key priority health problems to be used as case studies in this research. Despite nation-wide coverage of the National TB Control Program, TB remains a long standing problem across Pakistan, with key informants suggesting limited interest and action from BHU physicians related to TB program activities, especially the identification of new TB cases. Similarly, from January to April 2013, 192 measles cases were reported in the Attock district, largely at secondary health care facilities [21], with just three cases identified by the BHU physicians despite the fact that most of these cases were from geographical areas where they should have been reported by their respective BHU. Based on recommendations from the key informants, the study aims were developed:

- To document the flow of information^c^ on diagnosis and management of TB and measles cases in the PHC system of Pakistan;
- To describe the advice seeking behavior of physicians in situations with difficult to diagnose cases of TB and measles;
- To explore physicians’ satisfaction with their access to advice in difficult to diagnose cases of TB and measles;
- To identify and describe possible changes, if any, that physicians recommend to improve their access to advice in difficult to diagnose cases of TB and measles.

**Figure 1:**
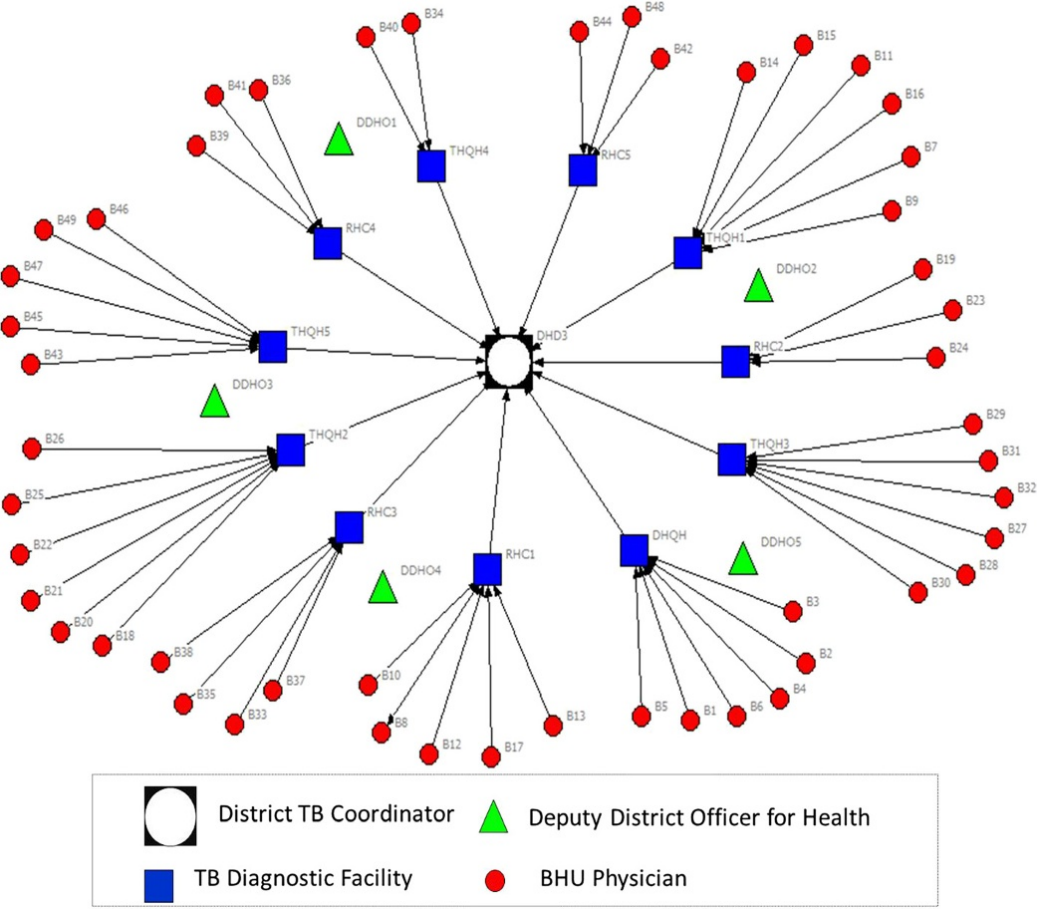
**Illustration of flow of information (advice on TB diagnosis) from district to BHU level under National TB Control Program.**

**Figure 2:**
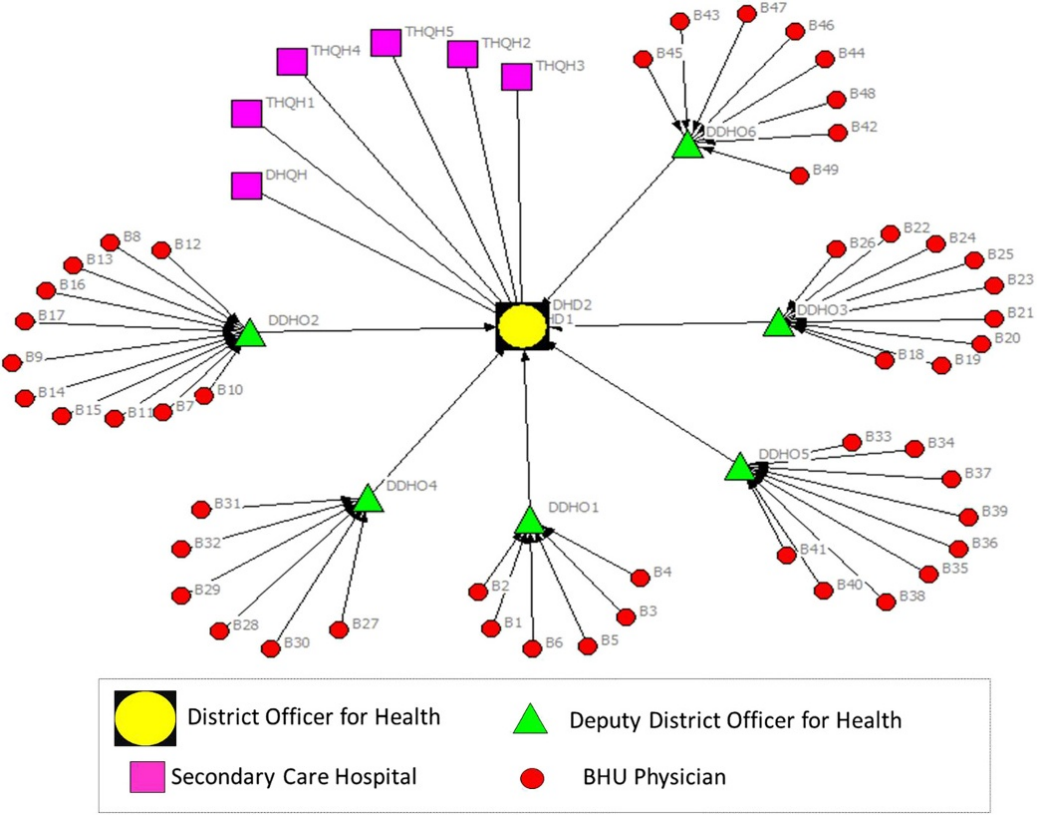
**Illustration of flow of information (advice on Measles diagnosis) from district to BHU level under Expanded Program on Immunization.**

In order to address these aims, with a cross-sectional study design and mixed-method approach, we employed three research methods comprising: i) mapping of existing information systems; ii) social network analysis of physician advice seeking behavior; and iii) key stakeholder interviews for in-depth understanding of physician experiences.Firstly, through documentary review (official memos for policy statements, job descriptions of doctors for roles and responsibilities, and training modules and guidelines for recordkeeping and reporting of suspected cases of TB and measles) and additional information obtained from five key informants, we mapped the existing flow of information system for assisting physicians in diagnosing TB and measles cases. Illustrations of formal information dissemination systems (Figures 1 and 2) were developed in the form of information flow charts showing the direction of flow of information and roles and responsibilities for providing information/feedback at various hierarchical levels. These illustrations were validated with the respective district health managers and BHU physicians for accuracy.

Secondly, a semi-structured questionnaire was used to conduct a survey for mapping social and professional networks among BHU physicians. We adapted Blanchet and James methodological approach for mapping and analysis of social networks [22, 23]. This approach comprises three stages: i) defining the list of actors and members of the network; ii) analyzing the relationships between actors; and iii) analyzing the structure and dynamics of social networks.

The survey questions were structured to identify whom each BHU physician had contacted for advice whenever faced with difficult to diagnose cases of TB and/or measles. All 49 physicians^d^ were invited to participate through an official intimation from the Executive District Officer for Health. With one exception, all physicians participated in the survey. Participation was voluntary and each physician completed his/her questionnaire in the presence of the principal investigator. In order to develop an egocentric network for analysis, each physician (ego) was asked to identify and name a person (alter) whom he/she had contacted to seek advice when faced with difficult to diagnose cases of TB and/or measles [24, 25]. As the scope of this research was limited to map the social networks from a BHU physician’s view only, alters were not contacted for confirmation. However, ‘name interpreter’ questions were asked of the physicians, designed to elicit further information about their respective alters, primarily covering their characteristics and relationship to the focal ego [25]. All different individual actors (egos and alters) were grouped based on their positioning (institutions/job titles) in the healthcare delivery system and placed under seven specific categories (Table 1).

**Table 1:** **Grouping of actors and institutions in seven categories**

No	Category	Number
1	District Coordinator for National TB Control Program	1
2	Deputy District Health Officers	5
3	TB Diagnostic Health Facilities	11
4	BHU Physicians	49
5	Consultants in Secondary Care Hospitals	1
6	Consultants in Tertiary Care Hospitals	3
7	Private Practitioners	1

**Figure 3:**
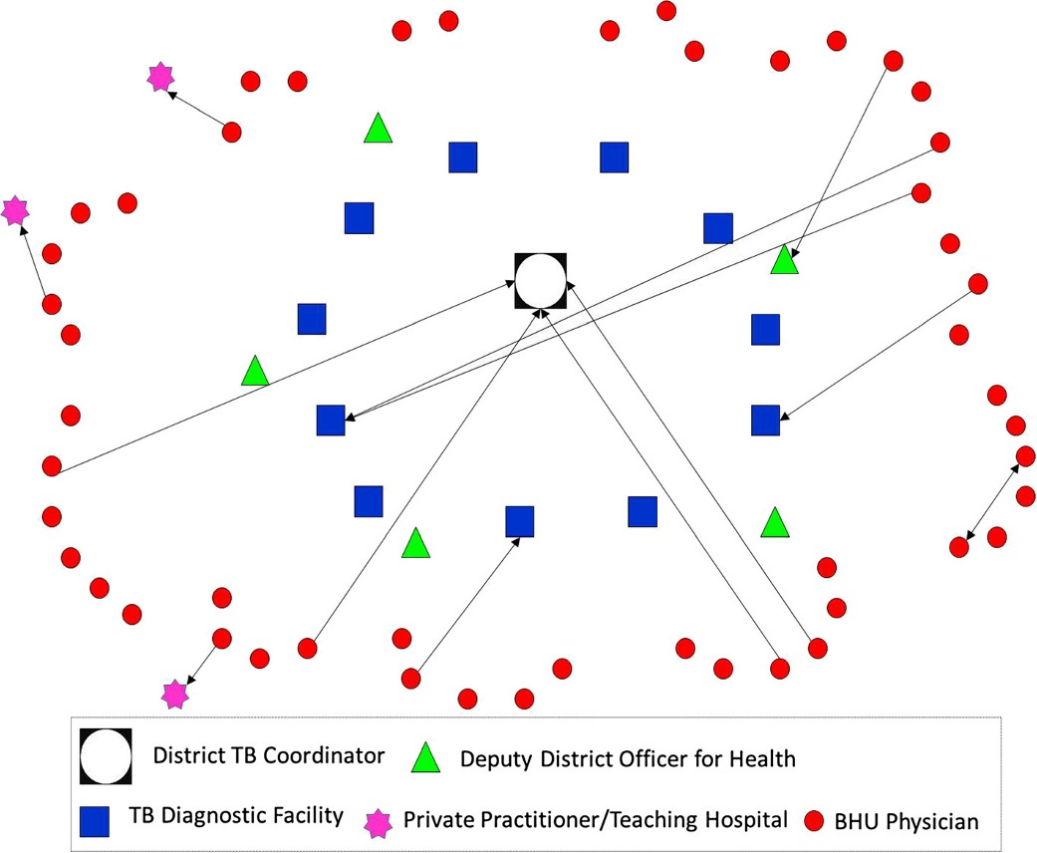
**Network of advice-seeking for difficult to diagnose cases of TB among BHU physicians.**

**Figure 4:**
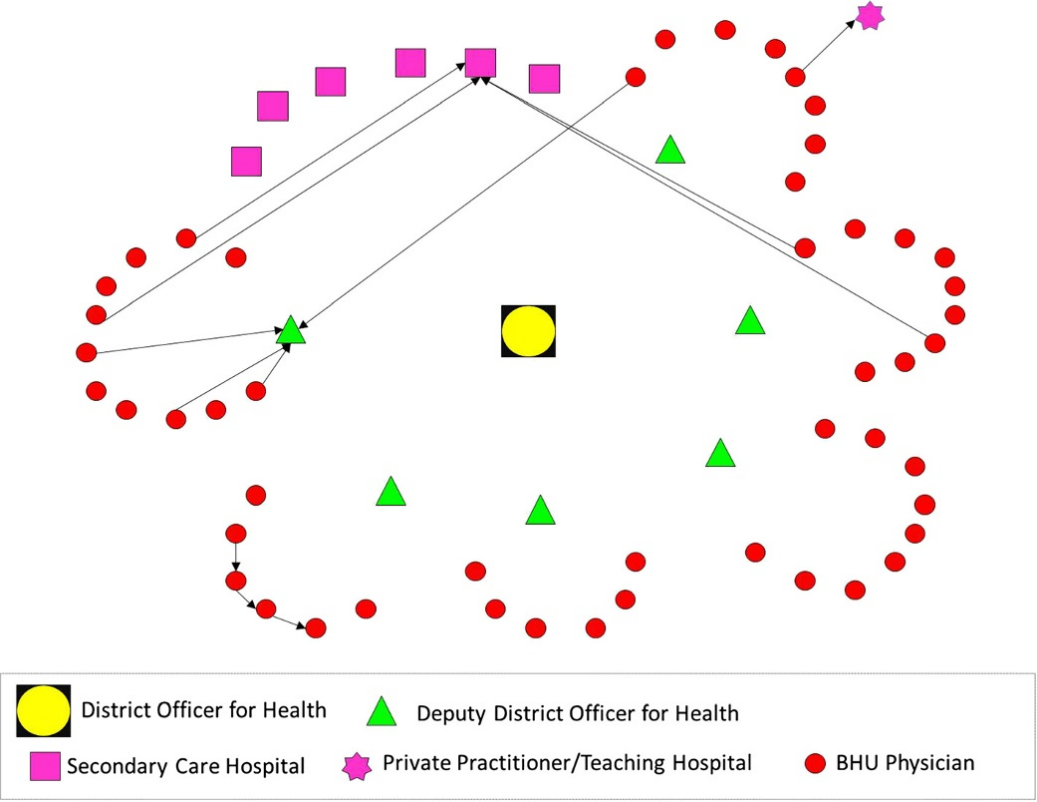
**Network of advice-seeking for difficult to diagnose cases of measles among BHU physicians.**

CINET software was used for generating directed-sociographs separately for TB and measles (Figures 3 and 4). A single-headed arrow indicates a directed-tie, from an ego at the tail and respective alter at the arrow-head, indicating the direction of advice seeking [25]. The different shapes represent various categories of actors (node) whereas the line (tie) connecting between two nodes indicates the presence of a relationship for advice seeking.

The analysis of sociographs indicated that a small number of physicians had sought advice when faced with a difficult to diagnose case of TB and measles (13 and 12 physicians, respectively). Given these findings, analyses of network properties such as betweenness, centrality, distance, and reachability were not performed. The sociographs provided visual illustrations of the existing network for advice seeking.

Thirdly, we conducted key stakeholder interviews in order to seek clarification and insights into the sociographs and to better understand the experiences of BHU physicians when they were faced with a difficult to diagnose case of TB and measles. Based on the analysis of the findings from sociographs, the BHU physicians were divided into three groups:

- Physicians who sought advice from a person designated by the district health department;
- Physicians who sought advice from someone other than a designated person;
- Physicians who did not seek advice from any other person.

This grouping provided the basis for selecting 11 study respondents for in-depth interviews (sampled from each of the above categories). The key domains explored during these interviews were reasons for seeking (or not seeking) advice, level of satisfaction under the present situation, and suggestions for improvement.

Interviews were digitally recorded and the average interview time was 20 minutes. All interviews were transcribed and content analysis was carried out by organizing coded data into categories, subthemes, and themes using an inductive process.

## Results

### Characteristics of BHU physicians

The study population of 48 BHU physicians comprised 41 males and 7 females. Average duration of government services was 6.7 years, ranging from 1 to 18 years. One-third (n = 16) of the study participants completed medical graduation overseas (mostly from Central Asian states). Further details are given in Table 2.

**Table 2:** **Characteristics of BHU physicians in Attock district**

Characteristics	Proportion (Number)
Attained medical education within Pakistan	66% (n = 32)
Employed as regular government employees	50% (n = 24)
Established private clinic besides government job	54% (n = 26)
Attended formal training on EPI* before joining service	66% (n = 32)
Attended formal training on TB DOTS** before joining service	85% (n = 41)

*EPI, Expanded Program on Immunization; **TB DOTS, TB Directly Observed Treatment Short-course.

### The formal system of information flow for diagnosing TB and measles cases

Under the National Tuberculosis Control Program (NTP), a District TB Coordinator (DTC) is responsible for administrative management of NTP activities at the district level. The DTC is also responsible for conducting trainings on Directly Observed Treatment Short-course (DOTS) strategy.

The District and Tehsil Headquarters Hospitals (DHQH & THQH)^e^ and Rural Health Centers^f^ are established as TB diagnostic centers with nomination of a TB focal person and upgradation of laboratory services for sputum and radiological examination service.

All 61 BHUs across the district are grouped in 11 clusters and every cluster is attached with a TB diagnostic center on the basis of geographical proximity. This administrative change has provided a formal link between primary and secondary health care facilities but only for matters related to TB. In their monthly meetings, BHU physicians are required to meet with the TB focal person at their respective TB diagnostic facility, who is expected to provide follow-up of patients referred by BHU physicians for confirmation of diagnosis, provide clinical advice and training, and advice on other programmatic issues.

The flow of information under the Expanded Program on Immunization (EPI) follows the organizational hierarchy of the district health care delivery system which is aligned with the geographical boundaries of district and sub-district levels. In comparison to NTP, the BHUs are not clustered around another hospital or higher level health facility, but rather the administrative office of the Deputy District Officer for Health (DDOH). In order to seek advice on issues related to the EPI (including measles), BHU physicians are expected to contact their respective DDOH (their immediate supervisor at Tehsil level). Further, in contrast to NTP, they do not have direct linkage with secondary care hospitals. The DDOH is also responsible for training on EPI, with facilitation from the District Officer for Health.

### Structure of professional and social networks for advice seeking

The structure of the social network for advice seeking reveals that 27% BHU physicians (n = 13) contacted a human information source when faced with a difficult to diagnose case of TB (Figure 3).

Ideally, as per the specified criteria established by the NTP [26], all were expected to contact the TB Focal Persons at their respective TB diagnostic centers (Figure 1); however, only two physicians contacted their designated source for advice. Among those that sought advice from an alternative source, four directly approached the DTC for an expert opinion, two consulted each other, whereas one sought advice from the respective DDOH. All contacted someone within the district except three who preferred to seek advice from a tertiary care hospital located externally to the district (Figure 3). The sociogram demonstrates a mixed pattern of advice seeking which is not consistent with expectations under the NTP.The structure of the social network for advice seeking for measles demonstrates that 25% of BHU physicians (n = 12) contacted a human information source when faced with a difficult to diagnose case of measles (Figure 4).

As per the departmental directions, all of the BHU physicians were expected to contact their respective DDHOs; however, only three physicians contacted their immediate supervisor. This finding was observed in one Tehsil only, with no physicians in the remaining five Tehsils seeking advice from their respective DDHOs. Four physicians preferred to contact a pediatrician in a single THQH. A similar number of physicians sought advice from another BHU physician. Only one physician contacted a private practitioner for advice. This pattern shows some differences from the advice-seeking network for TB because none of the physicians sought advice from outside the district.

The next section presents thematic analysis of the qualitative research findings from in-depth interviews.

### Qualitative findings from the in-depth interviews

The transcripts were coded and then organized into categories from which three subthemes and one main theme were identified. The analysis process is given in Table 3. The data from qualitative research findings are presented starting with the subthemes and their relation to the categories and concludes with how they contribute to the main theme.

**Table 3:** **Analysis process moving from categories to themes**

CATEGORIES	CATEGORIES	CATEGORIES
1. Physician considers first line officer incompetent	1. Specialist reachable over phone for advice if personally known	1. Lack of trust in organizational pathways for seeking information for patient care
2. Lack of support system for clinical guidance		
3. Lack of access to latest literature/research from the Health Department	2. Focus of departmental meetings on targets and not clinical guidance	2. Personal professional/social network (locally and distant) for patient care
4. Lack of functional referral system		
**SUBTHEME 1**	**SUBTHEME 2**	**SUBTHEME 3**
Lack of confidence in available resources	Unpredictable support	Reliance on personal (rather than organizational) resources to safeguard patients’ interests
**MAIN THEME**		
Formal organizational structures, including supervisory support and technical guidelines, not adequate		

### Subtheme 1: lack of confidence in available resources

All physicians had been working in BHUs for at least a year and were aware of the constraints and challenges associated with the BHU environment. They lacked confidence in the available resources for clinical guidance within the system.

#### Physician considers first line officer incompetent

Among those physicians who did not seek advice in relation to a complicated case of measles or TB, some suggested that the available experts within the district health care delivery system were not of the relevant field or lacked competence.

“*First of all, the doctors posted at TB diagnostic center should be competent enough. The doctor posted presently over there is not competent; I have no faith in him. Moreover, the position of medical specialist at THQH should be filled. Presently, a doctor is available at this hospital who has done his diploma in cardiology. Personally speaking, I don’t think he is competent enough to deal with TB patients.*”

Another physician reflected:

“*He is not an experienced person. Sometimes, he shows an X-ray to me to seek guidance on what to do further. For example, recently, he showed me an X-ray for advice whether that patient should be put on Streptomycin. I don’t consult him for advice because he is not that competent.*”

Physicians were aware of the limitations of their reporting officers or immediate supervisors and reported seeking advice from them in administrative issues only. Four physicians felt that they had not encountered any difficulty in diagnosing any difficulty and hence, did not seek advice from anyone.

#### Lack of support system for clinical guidance

For clinical advice related to measles, physicians reported trust in the opinion of pediatricians, while for TB, physicians reported willingness to contact the district TB coordinator. The need to improve access to those with relevant expertise was expressed, as was a formal mechanism within the PHC system for facilitating access to clinical guidance in a timely fashion. In addition, BHU physicians also reported being over-worked with organizing different campaigns and activities and were left with little time for patient care:

“*There should be a mechanism through which we can meet the specialists or have training session with them at least after every 2 to 3 months so that they know the BHU doctors and understand their problems. Although BHU doctors are performing their duties, it is a general perception that nothing is done at the level of BHUs. There is no one in a BHU after 11 am. These days, BHU doctors are heavily engaged in activities like Dengue control, measles vaccination campaign, and other official meetings. There is a single doctor posted at BHU level and when he is to remain away on account of all these official engagements, who will take care of the patients?”*

#### Lack of access to latest literature/research from health department

The main reliance of physicians for information was through other doctors and text-books:

“*If I require advice then I would prefer to reach out to my seniors because they have practical experience. I have never read research papers.*”

The internet was used to access information on complicated cases by some physicians; however, access to the internet was not available at every BHU. Other resource materials identified were pamphlets and brochures from representatives of pharmaceutical companies and handbooks on clinical guidelines. The health department did not provide such information:

“*There should be some system that BHU doctors remain updated with new developments. We will keep on making our personal efforts but there should be an official system, which should facilitate our access to updated information and research*.”

#### Lack of a functional referral system

There was also a lack of a functional referral system. As such, even when physicians consulted someone or referred them to another provider within the system, there was no mechanism in place to ensure feedback to the referring physician:

“*In case of diagnosis, I make a provisional diagnosis based on my clinical knowledge and books and then refer the patient accordingly. If the patients come back to my center afterwards, I get feedback from them.*”

Reiterating the non-functional feedback system, the physicians considered that it was more convenient to simply refer a suspected case of TB to a TB diagnostic facility without differentiating between a difficult or easy to diagnose case.

### Subtheme 2: unpredictable support

Interviewed physicians suggested that the health system worked in ‘spurts’, with the fluctuating functionality of the system physicians were driven to look for alternate sources when faced with complicated cases requiring advice.

#### Specialist reachable over phone for advice, if personally known

Specialists at times were available and reachable over the phone for advice on management/complications. This, however, was true in cases only when they personally knew the specialists and were close enough to them to call them.

“*I often contact Dr. A when I face problem in managing the treatment of TB patients. As far as history, symptoms and signs of TB are concerned, I think, I don’t have any problem with that. Further, 20–30% diagnosis is confirmed by the laboratory tests at the diagnostic center. Dr. A is our master trainer for TB program and I know him – that is why I often contact him for further advice.*”

Moreover, the referral center had the facilities for carrying out further tests.

“*The patient presented with enlarged and matted cervical lymph nodes. I talked to Dr. B about her and then she was referred to him because we thought she might require a biopsy. They have more facilities available at the THQH level.*”

#### Focus of departmental meetings on targets and not clinical guidance

Within the health department, most meetings focused on setting targets and achieving them. Clinical management was discussed only when a senior doctor was personally interested in clinical management and also competent to carry out such discussions. The support system to provide clinical guidance was non-existent. Thus, the system fluctuated as different doctors were appointed:

“*We participate in so many monthly-meetings because we have to remain in touch with our superiors and seniors. These meetings are not focused on diagnosis and patient management, rather the stress is on achieving our targets irrespective of whether these are achievable or not. Focus is more towards paperwork but not towards practical work.”*

### Subtheme 3: reliance on personal (rather than organizational) resources to safeguard patients’ interests

Physicians were motivated to seek advice and provide care to their patients. They realized the constraints within which they worked and had to provide best care to the patients.

#### Lack of trust in organizational pathways for seeking information for patient care

The physicians knew that there was no formal mechanism to seek advice within the system and there was a lack of trust in organizational information-seeking pathways as well. This was based on their own experience of not finding competent physicians posted at the higher health care facilities. Through their own professional network they sought advice:

“*Dr. C is a friend of mine and I can contact him easily.*”

One physician described how a personal experience with illness influenced his advice-seeking behavior and the need to safeguard patient’s interest to seek care:

“*Because I had suffered from the same situation myself; therefore, I directly contacted Dr. B. I had become so afraid of TB at that time because I was in my final medical year and exams were due in just a few months and my case was so much mismanaged. It is generally said that one becomes more sympathetic to a patient who shares the same experiences of suffering that a health provider has also gone through. That is why I referred this patient to a proper place so that they do not wander from place to place and get a trustworthy opinion.*”

The referring physicians indicated that there were internal contradictions in communicable disease reporting mechanisms: the reporting of cases of vaccine-preventable diseases, including measles, was received with some ambivalence by the higher authorities, given that they had been reporting high vaccine coverage for many years. The consequences for communicable disease surveillance were understandable, but undesirable; physicians were inclined not to document and report such cases. In complex cases of measles, it was easier to avoid negative consequences for the referring physician by not using formal reporting channels, but instead referring them directly to a pediatrician, outside the PHC system:

“*The more important thing is that every case of measles should be reported so that proper information is available in the health system. However, this is not the case in practice. Once you are discouraged by the high-ups that such cases are not to be reported, the doctor himself avoids reporting cases of measles so that he does not face any consequences. Being positioned at BHU level, we do not like to bear the responsibility. Therefore, if any case of measles turns up with or without complication, we do entertain him and refer him to a higher level of care but without the official reporting that is required in such a case.*”

#### Personal professional/social network (local and distant) for patient care

Many physicians used personal links to seek information for their patients. On encountering a complicated case, physicians reported reliance on other physicians/specialists they knew personally and could call over the phone to consult (even when specialists were present within the district health care system):

“*It depends upon the specialty or the type of the cases. For example, for Gynecology and Pediatrics I contact Dr. X (Gynecologist) whereas for pediatrics I contact Dr. Y (Pediatrician). For other cases I contact my colleagues and friends even outside the district because every specialist within the district cannot be contacted on personal relationship. For example, it is not possible to contact Dr. Z (Medical Specialist at DHQH) because he is not known to me.*”

### Main theme: formal organizational structures, including supervisory support and technical guidelines, not adequate

The main theme identified from the perspectives of interviewees illustrates how their work setting and information/advice-seeking patterns are driven by the constraints in organizational structures. It is difficult for BHU physicians to ensure comprehensive responsibility to their population if their efforts are not supported by specialized services available at secondary and tertiary levels of healthcare [1]. Getting their part of the system right does not help if other system components do not provide the required support [27]. Non-availability of competent supervisory staff, a focus on improving performance indicators rather than clinical guidance, and the lack of a functional referral system, collectively create an environment that is non-conducive for improving patient care. In the long term, physicians develop their own strategies to overcome these constraints. Their advice-seeking patterns largely depend on access to information systems and their contacts with colleagues within and outside the primary health care system. Non-responsiveness of the healthcare delivery system creates an environment where they work in isolation. Ultimately, it has negative consequences both for shouldering responsibilities and improving quality of care.

## Discussion

As noted by the WHO, the building blocks of health systems (service delivery; health workforce; information; medical products, vaccines and technologies; financing; and leadership and governance (stewardship)) while independently critical for system effectiveness, do not operate in isolation: complex relationships between the building blocks may help (or hinder) the overall ability of a system to use resources for improving health [12]. Human resources and information flow (two of the six building blocks), therefore, play critical roles in connecting sub-systems, promoting ongoing learning, and driving performance improvement. Examining how those within health systems share, access, and apply information is therefore important for better understanding how existing system structures and functions support or hinder learning and improvement.

Here, we discuss the implications of this study’s findings across four domains: system organizing, system networks, system dynamics, and system knowledge [13].

### System organizing

The designers of the NTP and EPI have achieved the program-specific institutional arrangements that they require at the sub-district level by clustering BHUs around TB diagnostic facilities and offices of the DDHOs, respectively, with additional administrative and managerial support provided from the district level. These institutional arrangements have provided an organizational skeleton for administration, training, and reporting through setting rules and regulations and assigning roles and responsibilities. However, these efforts have focused on establishing managerial control over program activities rather than nurturing a learning oriented environment that BHU physicians could rely on for advice, particularly when confronted by difficult to diagnose patient presentations. Consistent with previous reports from other jurisdictions [2, 20, 28], this study has identified non-availability of expertise and lack of a functional referral system as key deficiencies within the exiting PHC system, exacerbated by limited interconnections between BHU physicians and those providing higher, specialist levels of care. Moreover, even where designated centers do exist (such as in TB diagnostic facilities), BHU physicians rarely perceive these as authoritative or expert learning resources. Similarly, EPI line-managers (DDHOs) were infrequently contacted for advice, primarily due to a perceived lack of clinical knowledge related to the management of measles. The absence of a functional and reliable referral system further compounded this situation. Consequently, BHU physicians were more reliant upon their own knowledge and relationship to identify appropriate sources for advice.

### Systems networks

As noted, the findings from this study demonstrate that the information-seeking behavior of BHU physicians are diverse, driven by both the context and their network of available relationships, and do not follow formal organizational structures. The boundaries of information-seeking behavior do not appear to be limited by geography (district/province), type of health sector (public/private), or levels of healthcare (primary, secondary, and tertiary). Results from the qualitative investigation suggest that the presence of appropriate social ties (relationships) largely determine how physicians in this setting seek information from other human sources. In the Pakistani health system (as in other systems), specialist doctors are posted and available in the secondary and tertiary care hospitals with few formal linkages to the PHC infrastructure. Our study findings show that, while BHU physicians were aware of the human expertise available within and outside the district, their information-seeking behavior was largely influenced by their informal interactions and relationships with senior specialists.

Health systems are driven by humans and their interactions [29]. However, the existing PHC system in Pakistan does not appear to support or nurture relationships between junior and senior physicians. Participants in this study suggested a number of ways in which such connections may be fostered, such as greater in-service training events, more regular opportunities to work beside senior doctors, or even activities that build stronger connections between public and private sectors.

### System dynamics

Health systems are not static and adjust and readjust over time as contexts change, feedback is provided, and histories develop. The human instrument, unlike other resources, has the ability to cope and adjust to its environmental needs. Lack of response from higher authorities, discouraging attitudes, especially in the case of reporting vaccine preventable diseases (e.g., measles), and absence of feedback mechanisms, may demoralize frontline healthcare providers leading them to develop their own strategies outside formal accountability systems. Despite the limitations of the existing PHC system, a number of physicians in this study reported proactively using their professional linkages, experiences, and relationships to seek information and refer patients. However, there currently exists no process whereby referred patients may be followed-up in PHC centers. As a result, many physicians were not confident of the outcomes of referral under these circumstances, with patients often ‘lost in the system’. Although patient referral was occurring, long-serving public sector physicians were skeptical that a functional referral system could or would be created.

### Systems knowledge

While authority lies in knowledge [30], health system managers in this study were largely viewed as administrators lacking in clinical expertise. As a result, BHU physicians’ information-seeking behavior tended to be driven by perceptions of clinical expertise rather than hierarchical positioning or seniority. With inadequate use of resources to support physician training in TB DOTS and EPI prior to joining services, and a focus on management targets rather than clinical outcomes, BHU physicians developed their own learning and improvement strategies. Yet, without a supportive learning culture, many opportunities for better managing, sharing, and improving knowledge were missed, leading to isolation for many BHU physicians. In the absence of formal systems that meet their needs for clinical advice, individual physicians draw on their own networks of resources, and this in turn creates emergent organization that partly compensates for the gaps, but without addressing their causes.

### Limitations of the study

This study has a number of limitations. Firstly, the scope of the investigation was restricted to two specific diseases, TB and measles, in the context of a single district. Therefore, while the results are informative, they cannot be generalized to other diseases and geographical areas. Secondly, the network data were based upon the information provided by the PHC physicians. We did not contact the respective senior doctors for confirmation of the reported ties because it was beyond the scope of this research. Thirdly, this study has been conducted in public sector PHC facilities only and does not cover private sector PHC general practitioners who are a major source of service provision in Pakistan. However, as more than half of BHU physicians also work in the private sector, it is possible that findings may be similar across practice settings. Despite these limitations, the lessons learned could potentially be used in designing studies for providing comparative analyses in different contexts.

## Conclusions

Through the examples of TB and measles, this study has demonstrated how and why PHC physicians seek information when confronted with difficult to diagnose cases, and the challenges of creating learning systems that support continuous improvement. Given the number and diversity of patient presentations seen each day by PHC physicians, it is possible that there exists a more generalized need for high quality, reliable, and accessible information. Yet the absence of functional referral systems, limited effective linkages between PHC and higher levels of care, and a focus on programmatic targets rather than clinical care, have each contributed to the isolation of physicians and reactive information-seeking behavior. The advice-seeking behavior observed in this study may be explained by physician’s lack of confidence in available information resources, an unpredictable patient referral system, and a greater belief in personal rather than organizational resources for ensuring high quality patient care. Through interpreting these findings in partnership with those responsible for system design, it may be possible to assist provincial health departments in Pakistan to review the modalities of providing support to PHC physicians, especially in the country’s post-devolution phase. At this time, organizational and structural changes have a high potential of being entertained and implemented. The study findings underscore the need for a functional information system comprising context-sensitive knowledge management and translation opportunities for physicians working in PHC centers. Such an information system needs to link people and resources in ways that transcend geography and discipline, and that builds on existing expertise, interpersonal relationships, and trust.

## Endnotes

^a^Executive District Officer for Health, District Officer for Health and Deputy District Officer for Health.

^b^District Coordinator for National Tuberculosis Control Program, District Superintendent for Vaccination, Program Director for District Health Development Center, Coordinator for District Health Information System.

^c^For this paper, the flow of information is defined as formal mechanism of information exchange (reporting on case detection of TB and measles, through trainings, and advice seeking) in public sector health care delivery system at district level.

^d^Out of 61 BHUs in district Attock, physicians were appointed in 49 BHUs and the remaining positions were vacant at the time of survey.

^e^Secondary Care Health Facilities.

^f^A higher level PHC facility at a level between BHUs and secondary care health facilities.

## Abbreviations

BHU: Basic Health Unit
DDOH: Deputy District Health Officer
DHQH: District Head Quarter Hospital
DTC: District TB Coordinator
DOTS: Directly Observed Treatment short-course
EPI: Expanded Program on Immunization
NTP: National Tuberculosis Control Program
PHC: Primary Health Care
TB: Tuberculosis
THQH: Tehsil Headquarters Hospital.
